# Association of Schizoid and Schizotypal Personality disorder with violent crimes and homicides in Greek prisons

**DOI:** 10.1186/s12991-018-0204-4

**Published:** 2018-08-10

**Authors:** Athanasios Apostolopoulos, Ioannis Michopoulos, Ioannis Zachos, Emmanouil Rizos, Georgios Tzeferakos, Vasiliki Manthou, Charalambos Papageorgiou, Athanasios Douzenis

**Affiliations:** 1 2nd Psychiatric Department of the University of Athens, Attikon Hospital, Athens, Greece; 2 1st Psychiatric Department of the University of Athens, Aeginition Hospital, Athens, Greece; 3 Organization Against Drugs, Athens, Greece

**Keywords:** Personality disorders, Prisoners, Cluster A, Schizotypal, Schizoid

## Abstract

**Background:**

Personality disorders (PDs) have been associated with both violent crimes and homicides in many studies. The proportion of PDs among prisoners reaches up to 80%. For male prisoners, the most common PD in the literature is antisocial PD. The aim of this study was to investigate the association between PDs and violent crimes/homicides of male prisoners in Greece.

**Methods:**

A sample of 308 subjects was randomly selected from a population of 1300 male prisoners incarcerated in two Greek prisons, one urban and one rural. The presence of PDs was assessed using the Mini International Neuropsychiatric Interview (MINI) and the Personality Diagnostic Questionnaire-4 (PDQ-4). Using logistic regression models PD types and PD “Clusters” (independent variables) were associated with “violent/non-violent crimes” and “homicides/non homicides” (dependent variables).

**Results:**

“Cluster A” PDs (Paranoid, Schizoid, and Schizotypal) were diagnosed in 16.2%, “Cluster B” (Antisocial, Borderline, Histrionic, Narcissistic) in 66.9% and “Cluster C” (Obsessive–Compulsive, Dependent, Avoidant) in 2.9% of the studied population. Violent crimes and homicides were found significantly associated with “Cluster A” PDs (*p* = 0.022, *p* = 0.020). The odds ratio of committing violent crimes was 2.86 times higher for patients with “Cluster A” PDs than the ones without PDs. In addition, the odds ratio of committing homicides was 4.25 times higher for patients with “Cluster A” PDs. In separate analyses, the commitment of violent crimes as well as homicides, was significantly associated with Schizoid (*p* = 0.043, *p* = 0.020) and Schizotypal PD (*p* = 0.017, *p* = 0.030).

**Conclusions:**

The majority of prisoners was found to suffer from a PD, mainly the Antisocial “Cluster B”, but the commitment of violent crimes and homicides was significantly associated only with “Cluster A” PDs and specifically with Schizoid and Schizotypal PD.

## Background

According to *DSM-IV, the proportion of Personality Disorder* (PD) types in the general population is: 5.7% for “Cluster A” (Paranoid, Schizoid, Schizotypal), 1.5% for “Cluster B” (Antisocial, Borderline, Histrionic, Narcissistic) and 6.0% for “Cluster C” (Obsessive–Compulsive, Dependent, Avoidant) [1]. Furthermore, in 9.1% of the cases, two or more PDs co-exist [1]. Fourteen studies examined the risk of antisocial and violent behavior in 10,007 individuals with PDs, compared with over 12 million general population controls [2]. The results showed a substantially increased risk of violent outcomes in all PD types. Meta-analysis revealed that Antisocial PD and male gender were associated with the higher risks [2].

“Cluster B” PDs are affecting behavior and lifestyle and cause significant problems not only to the disordered individual but to society as well [3]. Among criminal offenders the proportion of Personality disorders is much higher [4] and can reach up to 80% in some studies [5]. Antisocial PD predominates among male offenders, while Borderline PD among female ones [6–8].

In a study by Riesco et al. [9] conducted in Spanish prisons, 91% of the prisoners had one or more PDs. Antisocial PD was diagnosed in 79% of the population while Paranoid PD and Borderline PD in 52% and 41%, respectively. Using the Mini International Neuropsychiatric Interview (MINI) and the Personality Diagnostic Questionnaire 4 (PDQ-4), Piselli et al. [10] found that the most frequent psychiatric disorder—in incarcerated offenders—was PD (51.9%). In a similar study by Coolidge et al. [11], 61% of prisoners had been diagnosed at least with one PD. Furthermore, Köhler et al. [12] found that the proportion of “Cluster B” PDs among incarcerated offenders was more than 62%. As mentioned before, Antisocial PD is found predominantly among male prisoners. This finding is supported by several studies: Kugu et al. reported a percentage of 48.6% [13], Naidoo and Mkize 46.1% [14] and Longato-Stadler et al. 56% [15]. In a systematic review of Fazel and Danesh [7]— 23,000 incarcerated individuals with more than 9000 violent prisoners included—PDs were diagnosed in 42% of the sample, among which 21% was Antisocial PD. Among prisoners in Greece, Fotiadou et al. found that the percentage of Antisocial PD was 37.5% [16].

However, Antisocial PD is not the only PD related to violent crimes. According to Fountoulakis et al. [17], Paranoid, Antisocial, Narcissistic, Borderline and Schizoid PDs (“Clusters A and B”) are all associated with violent crime. A significant association has been described, as well, between PDs of “Clusters A and B” and homicides. In case of homicide the most frequently encountered psychiatric diagnoses are PDs, drug addiction and alcohol abuse. More specifically, “Cluster B” PDs can be found in up to 60% of the cases. According to Crump et al. [18], people suffering from PDs and substance use disorders were more frequently convicted for homicide crimes. Richard-Devantoy et al. [8] claim, as well, that the association between comorbid Antisocial PD and alcoholism with murder is strong: Antisocial PD increased the risk of committing homicide 10 times compared to a Psychotic Disorder. In Finland, at least one diagnosis of PD was involved in 358 of the 593 homicides recorded from 1996 to 2004 [19].

As depicted, there is a much higher frequency of PDs in the prisons’ population compared to the general population. These disorders are predominantly “Cluster B” (especially Antisocial PD), followed by “Cluster A” PDs. Despite the fact that in the recent years there seems to be an increased scientific interest in studying and understanding the link between PDs and typology of criminal behavior, there are many contradictory findings that need clarification. In Greece, there have been no studies addressing these questions in prisoners.

This study was an attempt to record PDs among male prisoners in Greece and investigate the possible association between these disorders and violent offences and homicides.

## Method

### Study design and population

This is a cross-sectional study conducted in two Greek prisons; one urban (Korydallos) and one rural (Domokos). Data were collected from March 2012 till August 2013. The sample comprised of 308 male individuals randomly sampled from a total of 1300 prisoners, aged between 18 and 77 years. The sample included Greek and foreign prisoners who either had the Greek citizenship or could read and speak in Greek. Every third name of the registry of the prison was chosen. In 88 cases of unavailability/difficulties in understanding the language or denial (57 cases), the next prisoner was asked to participate in the study.

Inmates who were found to be suffering from psychotic disorders during the interviews were excluded from the study.

### Data collection

The psychiatric screening of prisoners was conducted with the Greek version 5.0.0 DSM-IV of Mini International Neuropsychiatric Interview (MINI) [20, 21] and the Greek version of Personality Diagnostic Questionnaire-4 (PDQ-4) [4, 22, 23]. The psychiatric examination was conducted by two psychiatrists. Initially demographics and other characteristics presented in Table 1 were recorded followed by a psychiatric assessment. The whole duration of the first interview was about 60 min. During the second interview, the PDQ-4 questionnaire was administered by the examiners, who were present till the end of the test (total duration about 60 min). Inmates who initially had shown mixed disorders or were not adequately assessed, were re-questioned (as suggested by the Personality Questionnaire manual) in order to conclude at one type of Personality Disorder. The third diagnostic interview’s duration was about 45 min.

**Table 1:** Demographics and other characteristics of prisoners in Greece (*n* = 308)

	*N* (%)
Age (*M*, SD years)	38.3 (10.8)
Nationality	
Greek	270 (87.7)
Other	38 (12.3)
Educational status	
Primary school	162 (52.6)
Middle school	125 (40.6)
College	10 (3.2)
University	11 (3.6)
Family status	
Married	93 (30.4)
Single	160 (52.3)
Widowed	6 (2.0)
Divorced	39 (12.7)
Separated	8 (2.6)
Children	123 (39.9)
Violence between parents	152 (49.4)
Relationship with father	
Good	192 (62.3)
Bad	63 (20.5)
Casual	53 (17.2)
Relationship with mother	
Good	245 (80.1)
Bad	14 (4.6)
Casual	47 (15.4)
Tobacco use	295 (95.8)
Alcohol use	272 (88.3)
Cannabis use	208 (67.5)
Drug use	179 (58.5)
Pyromania during childhood/puberty	204 (66.2)
Gang member	151 (49.0)
Animal abuse	201 (65.3)
Psycho traumatic event	126 (41.0)
Living status prior to imprisonment	
Alone	78 (25.4)
With family	98 (31.9)
With parents	65 (21.2)
Other	66 (21.5)
Soldiering	
Fulfilled	201 (65.3)
Dispensation	107 (34.7)
First imprisonment	158 (51.3)
Type of crime	
Violent	95 (30.8)
Non-violent	213 (69.2)

*M* mean value*, SD* standard deviation

Regarding the criminal record of the individuals assessed, the total number of incarcerations as well as their index offence that led to their imprisonment at the time of the study, were recorded. The assessing psychiatrists had no knowledge of the index offence during the initial interview. The offences that led to imprisonment were divided and categorized, according to the Greek Penal Code into violent (crimes against life, personal injuries, crimes against personal freedom, crimes against sexuality freedom, common danger crimes) and non-violent (crimes against ownership, debts to state, crimes related to drugs, crimes against property rights, crimes against honor, crimes related to marriage, crimes related to service, crimes related to currency). The crimes of homicide/attempted homicide were recorded separately from the other violent crimes and regardless the number of victims. Also for individuals that had multiple convictions, their most serious crime was recorded for the purpose of this study; e.g. for a conviction for both attempted murder and robbery, the individual was recorded under the category of “attempted murder”.

### Statistical analyses

Quantitative variables were expressed as mean values (*M*) and standard deviations (SD), while qualitative variables were expressed as absolute frequencies (*N*) and relative frequencies (%). For the comparison of proportions, Chi square and Fisher’s exact tests were used. Multiple logistic regression models with a stepwise method (*p* for entry 0.05, *p* for removal 0.10) were used in order to test whether PD types and PD “Clusters” were independently associated with violent crimes and homicides. PD types and PD “Clusters” were used as independent variables, whereas “violent/non-violent crimes” and “homicides–attempted homicides/non homicides” as dependent variables. Variables that regarded social, economic, demographic or clinical characteristics were not included in the analysis due to sample size. More statistical tests could have led to a lower study power. Adjusted odds ratios (OR) and the respective 95% confidence intervals (CI) were computed. Statistical significance was set at *p* ≤ 0.05. All reported *p* values are two-tailed and analyses were conducted using SPSS statistical software (version 19.0).

## Results

The study population consisted of 308 participants from a total of 1300 prisoners. Their mean age was 38.3 years (SD = 10.8). Almost half of the participants (51.3%) were imprisoned for the first time. For 30.8% of the participants conviction was a result of a violent crime. Homicide was recorded for 38 of the cases (12.3%). Homicides along with attempted homicides were recorded for 46 cases (14.9%). Demographics and other characteristics are presented in Table 1.

**Fig. 1:**
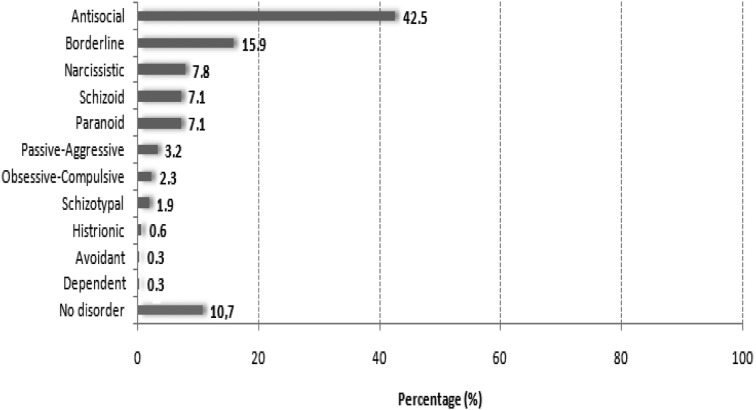
Personality Disorders’ percentages, among prisoners in Greece (*N* = 308)

PDs were diagnosed in 89% (*N* = 275) of the population. The types of PDs diagnosed are shown in Fig. 1. The most common PD was Antisocial (42.5%), followed by Borderline (15.9%), Narcissistic (7.8%), Schizoid (7.1%) and Paranoid (7.1%). For 10.7% (*N* = 33) of the participants no PD was diagnosed. “Cluster A” PDs were found in 16.2% of the participants, while “Clusters B and C” were found in 66.9% and 2.9% of the participants, respectively.

As extracted from Table 2, among prisoners with Antisocial, Borderline and Narcissistic Disorder, 77.1%, 65.3% and 58.3%, respectively, had been imprisoned for non-violent crimes. Nevertheless, most of the violent crimes (*N* = 30) as well as homicides/attempted homicides (*N* = 12) had been committed by individuals with Antisocial Disorder. Of those cases with Schizoid, Schizotypal and Paranoid disorder, 50.0%, 66.7% and 45.5%, respectively, had been incarcerated for violent crimes. On the other hand, 24.2% of violent crimes were committed by inmates with no diagnosis of a PD. Subjects of “Cluster A” PDs, in total, had committed 17 of the 46 homicides/attempted homicides.

**Table 2:** Differences in Personality disorder percentages among prisoners in Greece with violent crimes and homicides–attempted homicides (*N* = 308)

	Violent crimes	p*	Homicides/attempted homicides	p*
	*N* (%)		*N* (%)	
PD diagnosis				
Antisocial	30 (22.9)	0.870	12 (9.2)	0.608
Borderline	17 (34.7)	0.313	10 (20.4)	0.328
Narcissistic	10 (41.7)	0.162	1 (4.2)	0.385**
Paranoid	10 (45.5)	0.100	6 (27.3)	0.175**
Histrionic	1 (50.0)	0.454**	0 (0.0)	1.000**
Schizoid	11 (50.0)	0.049	8 (36.4)	0.047**
Schizotypal	4 (66.7)	0. 050**	3 (50.0)	0.026**
Obsessive–Compulsive	0 (0.0)	0. 309**	0 (0.0)	1.000**
Dependent	0 (0.0)	1.000**	0 (0.0)	1.000**
Avoidant	0 (0.0)	1.000**	0 (0.0)	1.000**
Passive–aggressive	4 (40.0)	0.330	2 (20.0)	0.611**
No PD	8 (24.2)		4 (12.1)	
PD Cluster				
A	25 (50.0)	0.019	17 (34.0)	0.025
B	58 (28.2)	0.641	23 (11.2)	0.774**
C	0 (0.0)	0.168	0 (0.0)	0.561**

*PD* Personal Disorder, *Cluster A PD* Paranoid/Schizoid/Schizotypal PD, *Cluster B PD* Antisocial/Borderline/Histrionic/Narcissistic PD, *Cluster C PD* Obsessive/Compulsive/Dependent/Avoidant PD

* Chi square test for the comparison with those without PD, ** Fisher’s exact test

Compared with prisoners without PD, the percentages of violent crimes and homicides/attempted homicides were significantly higher in those belonging to “Cluster A” and in those with Schizoid or Schizotypal PD alone (Table 2).

Multiple logistic regression analysis with “violent crimes/no violent crimes” as the dependent variable showed that the likelihood to commit a violence crime was significantly higher in “Cluster A” PDs (*p* = 0.022, OR = 2.86). When all the PD types were included in the model instead of PDs “Clusters”, it was found that Schizoid (*p* = 0.043, OR = 3.49) and Schizotypal (*p* = 0.017, OR = 10.5) PDs were significantly associated with a higher likelihood of violence crimes. Multiple logistic regression analysis with “homicides–attempted homicides/no homicides”, as dependent variable, had similar results. Prisoners of “Cluster A” (*p* = 0.020, OR = 4.25) and those with Schizoid (*p* = 0.020, OR = 5.26) or Schizotypal (*p* = 0.030, OR = 8.80) PDs had significantly increased likelihood for committing or attempting a homicide (Table 3).

**Table 3:** Multiple logistic regressions with “violent crimes” and “homicides–attempted homicides” as dependent variables in two Greek prisons (*N* = 308)

	OR (95% CI)	*p*
Violent crimes		
PD Clusters		
No PDs	1.00^a^	
A	2.86 (1.16–7.04)	0.022
B	1.91 (0.85–4.31)	0.118
C	–^b^	–
PD diagnosis		
No PDs	1.00^a^	
Antisocial	2.23 (0.83–6.05)	0.114
Borderline	1.91 (0.67–5.67)	0.229
Narcissistic	2.76 (0.85–8.97)	0.092
Paranoid	2.99 (0.91–9.81)	0.071
Histrionic	10.58 (0.53–210.99)	0.122
Schizoid	3.49 (1.04–11.73)	0.043
Schizotypal	10.50 (1.53–71.89)	0.017
Passive–aggressive	2.41 (0.51–11.49)	0.268
Homicides or attempted homicides		
PD Clusters		
No PDs	1.00^a^	
A	4.25 (1.26–14.31)	0.020
B	1.41 (0.42–4.70)	0.574
C	–^b^	–
PD diagnosis		
No PDs	1.00^a^	
Antisocial	1.35 (0.37–4.90)	0.645
Borderline	3.34 (0.87–12.79)	0.078
Narcissistic	0.36 (0.04–3.50)	0.379
Paranoid	2.96 (0.71–12.26)	0.135
Schizoid	5.26 (1.30–21.23)	0.020
Schizotypal	8.80 (1.23–62.78)	0.030
Passive–aggressive	2.00 (0.30–13.25)	0.474

*OR* adjusted odds ratio, *CI* confidence intervals, *PD* Personal Disorder, *Cluster A PD* Paranoid/Schizoid/Schizotypal PD, *Cluster B PD* Antisocial/Borderline/Histrionic/Narcissistic PD, *Cluster C PD* Obsessive/Compulsive/Dependent/Avoidant PD

^a^Indicates reference category, ^b^ could not be computed due to no distribution

## Discussion

### Personality disorders

The assessment of PDs, based on the DSM-IV classification and structured diagnostic instruments, is conflicting. It is possible individuals who meet the criteria for a particular Personality disorder meet as well the criteria for other Personality disorders. The new diagnostic approach in DSM-5 describes the Personality disorders as qualitatively distinct clinical syndromes. Nevertheless, in this study, diagnoses were based on MINI Interview and PDQ-4 Questionnaire because the use of the same instruments with other similar studies makes the results comparable and helps the scientific discussion on the association between violent crimes and psychiatric disorders.

PDs were diagnosed in the vast majority (89%) of the prisoners’ sample. The most common PD was Antisocial (42.5%). The likelihood of committing a violent crime or homicide–attempt homicide,was significantly greater among those with Schizoid or Schizotypal PD.

The high prevalence of psychopathology in the population of incarcerated offenders is well documented in the literature and also reflected in this study. PDs were the most common disorders among prison inmates in Italy [10]. At least one type of PD was diagnosed in 61% of a prison population sample according to Coolidge et al. [11]. Langeveld and Melhus reported that PDs were found in 80% of the prisoners. In the same study, antisocial PD was present in more than 60% of the study population [24]. Results from a systematic review of 62 studies with a total sample of 23,000 prisoners reported that 65% of the population had PDs, and 47% had Antisocial PD [7]. Similar results were reported in Greek populations by Fountoulakis et al. [17] and Fotiadou et al. [16].

In this study Antisocial PD was diagnosed in 42.5% of the participants, Borderline PD in 15.9% and Narcissistic PD in 7.8%. Histrionic PD was diagnosed only in 0.6% of the individuals. A definite dominance of “Cluster B” PDs was obvious which is in concordance with the literature. Köhler et al. found that the prevalence of “Cluster B” PDs in a sample of male incarcerated juvenile offenders in Germany was up to 62%. Findings from this study were very similar to our results: the proportion of “Cluster B” PDs was 66.9%, whilst “Cluster A” was 16.2% and “Cluster C” 2.9%.

### Personality disorders in relation to violent crimes

Of those diagnosed with Antisocial, Borderline and Narcissistic disorder in this study, 77.1%, 65.3% and 58.3%, respectively, had been imprisoned for non-violent crimes. Nevertheless, in absolute numbers, most of the violent offenses had been committed by inmates presenting Antisocial Disorder. However, a significant association of violent crimes and “Cluster B” PDs has not been established. According to Palmstierna, Antisocial PD and antisocial personality traits are connected with violence [25]. Similarly Pondé et al. and González et al. suggest a strong association of Antisocial and Borderline PDs with violent crime [26, 27].

High incidence of “Cluster B” PDs is often seen in the literature, although higher rates of “Cluster A” disorders have also been reported in prison’s populations, usually associated with a high prevalence of Paranoid PD [9]. In this study, 16.2% of prisoners had “Cluster A” PDs; 1.9% Schizotypal; 7.1% Schizoid, and 7.1% Paranoid PD. Diagnosis with “Cluster A” disorders had an association with the commitment of violent crimes. Of those diagnosed with Schizoid, Schizotypal and Paranoid disorder 50.0%, 66.7% and 45.5%, respectively, have been incarcerated for violent crimes. These results are in concordance with Esbec and Echeburúa who reported that increased symptoms of DSM-IV “Cluster A” or “Cluster B” PDs, such as paranoid, narcissistic and antisocial symptoms are significantly associated with violence [28]. Accordingly, Mouilso and Calhoun reported a strong association of Narcissistic PD with sexual assault [29]. On the other hand, increased borderline personality tendencies have been reported in female sexual abusers [30]. Serial offenders as well are more likely to have Narcissistic, Schizoid and/or Obsessive–Compulsive traits. They are also more likely to engage in sexual masochism, partialism, homosexual paedophilia, exhibitionism and/or voyeurism, according to Chan et al. [31]. In addition, Pulay et al. reported an association between Schizoid, Paranoid and Obsessive–Compulsive PDs with violent behavior [32]. Another study by Haller also suggested a significant association of paranoid disturbance with violent crimes [33]. Schizoid PD is related as well with features of psychopathy and Antisocial personality according to Kosson et al. [34]. Loza and Hanna argue that an association exists between Schizoid PD and violent acts [35]. This study found a significant relation only of “Cluster A” disorders with violent offenses.

“Cluster C” disorders accounted for a minority of cases; individuals with Obsessive–Compulsive PD were only 2.3% of the study sample, whereas Dependent PD and Avoidant PD were few (~ 0.3%). This is in concordance with findings from Finland; “Cluster C” disorders comprised 3.5% of the entire sample of 593 offenders [19]. However, in contrast with the aforementioned studies, there were no violent crimes committed by offenders with “Cluster C” PD in this study, possibly due to the very small proportion of this PD “Cluster” in the studied population.

As presented in logistic regressions’ results (Table 3), prisoners with any type of PD have greater likelihood (OR) of committing a violent crime. Nevertheless, violent crimes were associated significantly (*p* ≤ 0.05) only with Schizotypal and Schizoid PD likely because the comparison group was composed by another type of criminals, instead of being composed by the general population.

### Diagnosis with Personality disorders and association with homicides–attempted homicides

PDs have been found as principal or secondary diagnosis between homicides and attempted homicides offenders [37]. According to Pera and Dailliet, in a sample of 32 Belgian offenders 17 had an Antisocial PD, 8 a Borderline PD, 4 a Paranoid, and 2 a Schizoid PD [38]. Also, in a sample of 36 convicted Jamaican murderers 66% had an Antisocial PD [39]. Antisocial PD and substance use disorders were the most prevalent psychiatric diagnoses among prisoners that had committed or attempted homicide, as suggested by Kugu et al. [13].

Concerning sexual murderers, they are often diagnosed with a PD, especially with Schizoid PD [40]. Myers and Monaco and others also found an association of Sadistic PD (as described in DSM-IV) with sexual homicide [41, 42]. Concerning serial homicide offenders, they are more likely to have Narcissistic, Schizoid and/or Obsessive–Compulsive traits according to Chan et al. [31]. Loza and Hanna reported, as well, an association between Schizoid PD and violent homicidal behavior [35]. Analysis of case reports by Jeffrey Dahmer and Dennis Nilsen underlined an association between schizoid personality traits with violent antisocial behavior [43].

In children, schizotypal features elicit victimization from other children, which in turn predisposes to reactive retaliatory aggression [44]. Lam et al. found that schizotypal personality traits (schizotypy) are associated with antisocial behavior [45]. This relation is replicated in the literature linking Schizotypal Disorder with antisocial behavior and violent crime [45].

Regarding homicide–attempted homicide in this study, the majority was committed by individuals suffering from Antisocial PD. Subjects of “Cluster A” PDs, in total, had committed 17 of the 46 crimes of this type. These results are in contrast to Keue and Borchard [36] and Laajalo et al. [19] studies that found no association between disorders of “Cluster A” and homicides. Prisoners of this study, with disorders of “Cluster A”, were 4.25 times more likely to commit murder, while individuals with “Cluster B” disorders were 1.41 times more likely to commit the particular offense, compared with subjects without PD. Specifically for Antisocial PD odds ratio was 1.35, for Borderline PD was 3.34, for Narcissistic PD was 0.36, for Paranoid PD was 2.96, for Schizoid PD was 5.26 and for Schizotypal PD was 8.80, compared with subjects without PD. However, the committed homicide–attempted homicide was significantly associated with only Schizotypal and Schizoid PD.

Possibly, there is a neurobiological contribution to the association between Schizoid and Schizotypal PD and commitment of homicides or violence crimes. In literature, Schizotypal traits are associated with high hostility levels [46]. According to Raine et al. schizotypy was associated with total and reactive aggression but not with proactive aggression [44]. Sexual murderers are often diagnosed with a Schizoid PD [40]. Lam et al. [45] suggested that orbitofrontal cortex gray matter mediated the effect of schizotypy on antisocial behavior by 53.5%. On the other hand, this association was not significant for prefrontal cortex sub-regions. These findings highlight the specificity of the orbitofrontal cortex in understanding the schizotypy–antisocial behavior relationship. A link between Schizoid PD and Schizotypal PD was suggested by Via et al. [47]. According to them, persons with Schizoid PD–Schizotypal PD have greater bilateral white matter volume in the superior part of the corona radiata, close to motor/premotor regions, compared to healthy controls.

Schug et al. reported that reduced skin conductance orienting to neutral tones may reflect a neurocognitive risk factor, for both Antisocial and Schizotypal PDs that indirectly reflects a common neural substrate to these disorders [48]. Other researchers reported that individuals with Schizotypal PD display heightened activation in the neural circuitry, involved in reward and decision making when viewing biological motion stimuli in addition to a positive correlation between increased blood oxygenation level-dependent, signal responses related to biological motions and clinical symptoms [49]. These findings suggest that enhanced responses arise within the reward network for individuals with Schizotypal PD and are possibly related to the “peculiar” ways that individuals with Schizotypal disorder behave in social contexts. It might be the “unemotional and cold part” of individuals with Schizoid and Schizotypal PD that contributes to the increased occurrence of “lethal violence”.

This study addresses certain limitations. Although the number of the participants is quite large, a bigger sample of individuals would have enhanced our results. For example, association of Paranoid (“Cluster A”) and Narcissistic (“Cluster B”) PDs with violent crimes was slightly not statistically significant (*p* values were 0.09 and 0.07, respectively). Another limitation is that issues of “free” psychopathology and counter-transference were not addressed in the initial protocol. It is also likely that Personality disorders have been overestimated in this study as well as in studies using structured diagnostic instruments. Much higher prevalence possibly has been reported compared to clinically based studies.

## Conclusions

Most of the Greek prison inmates were diagnosed with a PD with a clear predominance of Antisocial PD. Nevertheless, significant associations of violent crimes and the offense of homicides–attempted homicides were found for the Schizoid and Schizotypal PD. No association of “Cluster B” or “Cluster C” PDs with violent crimes was elicited. This study—conducted for the first time in a Greek prisoners’ population—provides evidence that murders and attempted murders are strongly associated with Schizoid and Schizotypal PDs. Further research is necessary to study in more depth the possible association of PDs—especially of the “Cluster A”—and the type of crime in Greece. Also, studies with larger samples and higher statistical power could investigate the role of other social, economic, demographic and clinical determinants in appearance of violent crimes and committed or attempted homicides.

## Abbreviations

PDs: Personality disorders
MINI: Mini International Neuropsychiatric Interview
PDQ-4: Personality Diagnostic Questionnaire-4
M: mean
N: absolute frequencies
SD: standard deviation
OR: odds ratios
CI: confidence intervals
